# Impact of COVID-19 Lockdown on NO_2_ Pollution and the Associated Health Burden in China: A Comparison of Different Approaches

**DOI:** 10.3390/toxics12080580

**Published:** 2024-08-10

**Authors:** Zhiyuan Li

**Affiliations:** School of Public Health (Shenzhen), Sun Yat-sen University, Guangzhou 510275, China; zhiyuan.li@connect.ust.hk; Tel.: +86-13058180792

**Keywords:** COVID-19 lockdown, NO_2_ pollution, machine learning, random forest, China

## Abstract

So far, a large number of studies have quantified the effect of COVID-19 lockdown measures on air quality in different countries worldwide. However, few studies have compared the influence of different approaches on the estimation results. The present study aimed to utilize a random forest machine learning approach as well as a difference-to-difference approach to explore the effect of lockdown policy on nitrogen dioxide (NO_2_) concentration during COVID-19 outbreak period in mainland China. Datasets from 2017 to 2019 were adopted to establish the random forest models, which were then applied to predict the NO_2_ concentrations in 2020, representing a scenario without the lockdown effect. The results showed that random forest models achieved remarkable predictive accuracy for predicting NO_2_ concentrations, with index of agreement values ranging between 0.34 and 0.76. Compared with the modelled NO_2_ concentrations, on average, the observed NO_2_ concentrations decreased by approximately 16 µg/m^3^ in the lockdown period in 2020. The difference-to-difference approach tended to underestimate the influence of COVID-19 lockdown measures. Due to the improvement of NO_2_ pollution, around 3722 non-accidental premature deaths were avoided in the studied population. The presented machine learning modelling framework has a great potential to be transferred to other short-term events with abrupt pollutant emission changes.

## 1. Introduction

Nitrogen dioxide (NO_2_) is a typical toxic air pollutant and a precursor to ozone (O_3_) and secondary aerosols [1,2,3]. Exposure to elevated NO_2_ concentrations can cause a number of adverse health effects, including respiratory diseases, lung cancer, and even premature deaths [2,4]. In urban areas, the main sources of NO_2_ include fossil fuel combustion and traffic emissions [5,6]. Thus, NO_2_ is an effective indicator of traffic emissions in cities. The trend of NO_2_ concentration can be used to examine the effectiveness of air quality management and the influence of abrupt pollutant emission changes [4,7].

The novel coronavirus disease (COVID-19) broke out in the city of Wuhan, Hubei province, China, in late December 2019 [8,9]. By 23 January 2020, the Chinese authorities undertook stringent traffic restrictions and self-quarantine measures to control the spread of COVID-19 [10,11]. This lockdown policy lasted until the end of February in regions outside Hubei province and until the start of April in Hubei province [12]. Scientific results showed that air pollutant concentrations dropped dramatically in China with the implementation of these lockdown measures [5,9,10,13]. For instance, Chu et al. [10] found that the average NO_2_ concentration decreased by 53%, 50%, and 30% in Wuhan city, Hubei province (Wuhan city excluded), and China (Hubei province excluded), respectively, during the lockdown period of early 2020 compared with levels from the same period in 2019. In addition, Wang et al. [9] reported that the air quality index and concentrations of NO_2_, fine particulate matter (PM_2.5_), and carbon monoxide (CO) reduced by 15%, 38%, 22%, and 20% in Northern China, respectively, due to the lockdown measures. Furthermore, Cai et al. [14] estimated that a 25.3%–48.8% drop in the concentrations of NO_2_ has been observed in the four metropolises (Shanghai, Tianjin, Guangzhou, and Wuhan) during the quarantine period compared with the same period in 2018–2019.

With the spread of COVID-19 around the world, a large number of countries took a series of lockdown measures to prevent the pandemic. These lockdown policies reduced air pollutant concentrations around the world, especially in urban areas where intensive human activities stopped [15,16,17,18,19,20,21,22,23]. For instance, owing to the COVID-19 lockdown, there was a reduction in NO_2_ concentration by 50% and 62%, respectively, in Madrid and Barcelona, Spain [15]. Moreover, Liu et al. [24] reported that California had a 38%, 49%, and 31% drop in the concentrations of NO_2_, CO, and PM_2.5_ during the lockdown period (19 March–7 May) compared to the pre-lockdown period (26 January–18 March) in 2020. In addition, Filonchyk et al. [25] quantified that the NO_2_ levels in Poland were reduced by −25% and −19%, respectively, as compared to the same period in 2018 and 2019, due to lockdown measures. Several studies documented that there was a slow increase in air pollution with the gradual return of human activities after the lockdown [1,13,14].

The evaluation of the COVID-19 lockdown measures can provide valuable insights into the adjustment for a better policy implementation and for the design of similar policies in the future or in other regions [9,26,27]. The research methods of satellite remote sensing, atmospheric chemical transport models (ACTMs), statistical analysis models, machine learning algorithms, and others were intensively used to conduct evaluations of the COVID-19 lockdown measures and meteorology on changes in air quality [1,5,11,27,28,29,30,31]. Among these research methods, the machine learning approach, which has been reported to have high prediction accuracy and strong non-linear relationship modelling capacity [12,13,26,29], has great potential for understanding the true effect of influencing factors on air quality. Up until now, to the best of our knowledge, there are very few studies utilizing the machine learning approach against other approaches to examine the effect of COVID-19 lockdown policies on air quality in the whole country of China [13,26]. It is therefore essential to reveal the evolution of air pollution and human health due to the lockdown policies using the machine learning approach, as well as other approaches.

The main research objective of the present work is to compare two approaches, namely the machine learning and difference-to-difference approaches, to quantify the effect of the COVID-19 lockdown policies on NO_2_ pollution during the COVID-19 outbreak period in mainland China. Section 2 presents the NO_2_ and meteorology measurement data and the methods used for the data analysis. The results and discussion, including the established random forest models, the spatiotemporal variability of the COVID-19 lockdown effect on NO_2_ pollution, and the health benefits from NO_2_ reduction, are summarized in Section 3. The conclusion section summarizes the findings and implications gained from the analysis on the effect of the COVID-19 lockdown policy.

## 2. Materials and Methods

### 2.1. Data Collection and Processing

In this study, 31 provincial capital cities in mainland China are included, with Hong Kong, Taipei of Taiwan, and Macau not included due to different management policies during the COVID-19 outbreak period. The period of 1 December 2019 to 30 April 2020 is the study period, while the same time periods of the previous three years, i.e., 1 December 2016 to 30 April 2017, 1 December 2017 to 30 April 2018, and 1 December 2018 to 30 April 2019, are the reference periods for the development of random forest machine learning models. The time periods of lockdown policies for the included cities were summarized from the central, provincial, and local government websites. The period before the COVID-19 lockdown period (pre-lockdown) is defined as 1 December 2019 to 22 January 2020, which is the date when the city lockdown started in Wuhan; the COVID-19 lockdown period (lockdown) is defined as the city lockdown period of 23 January to 18 March 2020; and the period after the COVID-19 lockdown period (post-lockdown) is defined as the date when the city re-opened, which is 19 March 2020 to 30 April 2020.

NO_2_ was selected as the studied pollutant because it is significantly and directly affected by the lockdown activities [1]. The daily mean NO_2_ measurement data were downloaded from the China National Environmental Monitoring Center (Available online: http://beijingair.sinaapp.com/ (accessed on 15 October 2023)). The meteorological data (wind direction, wind speed, temperature, and relative humidity) measured at the nearest weather stations were collected from the Global Historical Climatology Network Daily (Available online: https://www.ncdc.noaa.gov/ghcn-daily-description (accessed on 15 October 2023)) and used as the meteorological conditions. Following previous studies [2,32], the air quality monitoring stations were matched with the nearest weather stations to gain the related meteorological parameters.

### 2.2. Evaluation Approaches

#### 2.2.1. Random Forest Models

Daily average values of NO_2_ concentration at 31 provincial capital cities were the dependent variables. The potential predictor variables were meteorological and environmental variables and time variables. The meteorological conditions affect the accumulation and transport of air pollution, while the time variables served as the major proxy of emissions [9,33,34,35]: the year represents the change in emission factors, day_julian and weekday show the trends of emissions during a year and a week, and day_lunar represents the number of days referring to the first day of the Chinese Lunar New Year holiday (e.g., 27 January was the first day of the holiday in 2017; therefore, day_lunar for 26–28 January in 2017 was −1, 0, 1, respectively) [32,36].

The random forest model [13,37], which is commonly adopted in previous air quality prediction studies, was used in this work. A detailed description of the random forest approach can be found in Grange and Carslaw [34] and Li et al. [38]. A combination of ntree values ranging between 50, 100, 200, 500, and 1000, and mtry values ranging between 2, 3, 4, 5, 6, and 7 were used to evaluate the model performance. After comparison, the ntree value of 500 and mtry value of 4 were selected to develop the final models. For each provincial capital city, one random forest model was established over 2017–2019, and 31 random forest models were established in total. The established random forest model was used to predict the NO_2_ concentrations for the 2020 COVID-19 lockdown period. For validation, the datasets of two years from the 2017–2019 period were used to establish the model, and the dataset of the remaining year was used to compare with predictions. This process was repeated three times. In addition, the established 2017–2019 random forest model was used to predict NO_2_ concentrations for the pre-lockdown and post-lockdown period in 2020, and the comparison between predictions and observations of NO_2_ concentrations was conducted to further validate the models. 

The commonly used statistical parameters, including the coefficients of determination (R^2^), mean bias error (MBE), normalized mean bias error (NMBE), mean absolute error (MAE), normalized mean absolute error (NMAE), root mean squared error (RMSE), and normalized root mean squared error (NRMSE) [13,38,39,40,41] were employed to evaluate the developed random forest models. The statistical analysis was performed using R software, version 4.0.3 (R Foundation for Statistical Computing, Vienna, Austria).

#### 2.2.2. A Difference-to-Difference Approach

For the comparison with the results using the random forest models, we also applied a difference-to-difference approach [42] to quantify the effect of the COVID-19 lockdown on NO_2_ pollution.

(1) $$C_{d,\mathrm{BLP}}={\bar{C}}_{2020,\mathrm{BLP}}-{\bar{C}}_{2017-2019,\mathrm{BLP}}$$

(2) $$C_{d,\mathrm{ALP}}={\bar{C}}_{2020,\mathrm{ALP}}-{\bar{C}}_{2017-2019,\mathrm{ALP}}$$

(3) $$C_{d,\mathrm{DLP}}={\bar{C}}_{2020,\mathrm{DLP}}-{\bar{C}}_{2017-2019,\mathrm{DLP}}$$

(4) $$C_{\mathrm{adj}\_d,\mathrm{DLP}}=C_{d,\mathrm{DLP}}-{\;C}_{d,\mathrm{BLP}}$$

where $C_{d,\mathrm{BLP}}$, $C_{d,\mathrm{DLP}}$, and $C_{d,\mathrm{ALP}}$ represent the pollutant concentration difference of the 2020 concentration against the 2017–2019 mean concentration of the before lockdown period (BLP), during lockdown period (DLP), and after lockdown period (ALP), respectively. $C_{\mathrm{adj}\_d,\mathrm{DLP}}$ represents the adjusted pollutant concentration difference of the 2020 concentration against the 2017–2019 mean concentration DLP, with the concentration difference of the BLP removed. ${\bar{C}}_{2020,\mathrm{BLP}}$, ${\bar{C}}_{2020,\mathrm{DLP}}$, and ${\bar{C}}_{2020,\mathrm{ALP}}$ are the mean pollutant concentrations of the BLP, DLP, and ALP in 2020. ${\bar{C}}_{2017-2019,\mathrm{BLP}}$, ${\bar{C}}_{2017-2019,\mathrm{DLP}}$, and ${\bar{C}}_{2017-2019,\mathrm{ALP}}$ are the 2017–2019 mean pollutant concentrations of the BLP, DLP, and ALP, respectively.

### 2.3. NO_2_ Concentration Changes in 2020 Due to the COVID-19 Pandemic

The values of NO_2_ concentrations of the studied cities over the 2020 lockdown period were compared with the random forest model predicted values (representing no lockdown scenarios). The spatial distribution of changes in NO_2_ concentrations owing to the lockdown was visualized using ArcGIS software v10.6 (Esri, New York, NY, USA). The time series plots of observations and predictions on NO_2_ concentrations for five example cities, including Beijing (the capital city, located in Northern China), Shanghai (located in Eastern China), Guangzhou (located in Southern China), Wuhan (located in Central China and the center of COVID-19 outbreak in China), and Xi’an (located in Western China) in 2020, were presented. 

### 2.4. Health Impact Assessment

The health benefits due to the reduction in NO_2_ concentrations during the COVID-19 lockdown period were estimated using the classic health impact assessment method [43,44].

(5) $$RR=exp^{\beta \Delta c}$$

(6) $$AF=\frac{RR-1}{RR}=1-exp^{-\beta \Delta c}$$

(7) $$\Delta Mort=y_{0}(1-exp^{-\beta \Delta c})Pop$$

where ΔMort is the health benefits in the mortality or morbidity based on NO_2_ reductions, β is the cause-specific coefficient of the concentration–response functions, $y_{0}$ is the mortality rate in the reference scenario, Pop is the population for each studied city, *Δ*c is the NO_2_ reduction during the COVID-19 lockdown period, RR refers to the relative risk of NO_2_ pollution, and AF is the fraction of the disease burden attributable to the risk factor. In this work, β values of 0.009, 0.009, and 0.012 were adopted for estimating the avoided health burden from non-accidental diseases, cardiovascular diseases, and respiratory diseases, respectively [45]. AF was multiplied by the daily cause-specific number of deaths and the total number of days during the COVID-19 lockdown period.

## 3. Results and Discussion

### 3.1. Development and Validation of Random Forest Models

**Figure 1:**
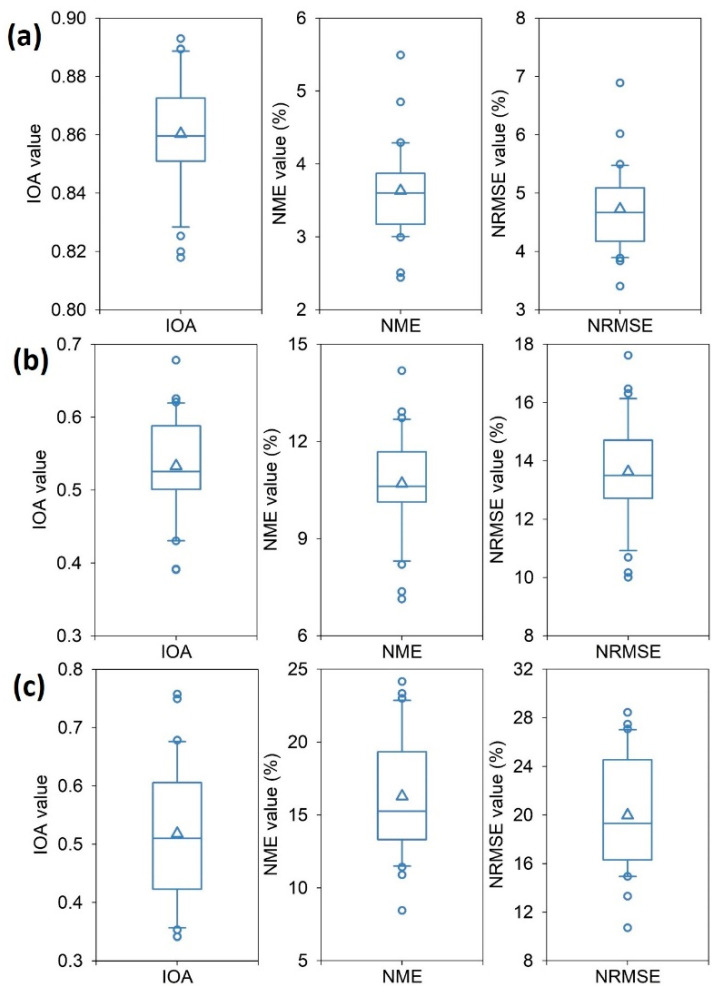
The box plots of IOA, NME, and NRMSE values for the (**a**) development, (**b**) three-year cross-validation, and (**c**) 2020 NO_2_ concentration predictions. The triangle in each box is the mean value, the solid line is the median value, the box extends from 25th to 75th percentile, the whiskers (error bars) below and above the box are the 10th and 90th percentiles, and the below and upper cycle symbols are the outliers. (For interpretation of the references to color in this figure legend, the reader is referred to the Web version of this article).

A summary of statistical metrics for the development, three-year cross-validation, and 2020 predictions of the random forest models is shown in Figure 1. For the development of random forest models, the IOA values ranged between 0.85 and 0.93, with an average value of 0.91. The NME and NRMSE values ranged between 2% and 6%, and between 3% and 7%, respectively (Figure 1a). The overall conclusion is that the established random forest models achieved quite high predictive performance.

In terms of the three-year cross-validation, the IOA, NME, and NRMSE values varied between 0.39 and 0.68, between 8% and 15%, and between 10% and 18%, respectively (Figure 1b). The average values for IOA, NME, and NRMSE were 0.58, 13%, and 14%, respectively, when conducting the cross-validation, which are still within the threshold values for modelling [39,46].

To further evaluate the performance of the established random forest models, the predicted NO_2_ concentrations and corresponding observations during the pre-lockdown period (i.e., 1 December 2019 to 22 January 2020) were compared. The IOA, NME, and NRMSE values ranged between 0.34 and 0.76, between 8% and 15%, and between 10% and 18%, respectively (Figure 1c). The values of these statistical metrics all met the criteria thresholds recommended in previous studies [2,39,46]. The consistency of NO_2_ predictions and observations during the pre-lockdown period gives the authors confidence to infer that the differences between NO_2_ predictions and observations during COVID-19 lockdown period are due to the COVID-19 lockdown measures, such as the shutdown of manufacturing and heavy industry factories, and the restriction of traffic flows.

The statistical metrics of the established random forest models varied among different cities to some extent (Figure 1). In addition, the feature importance of these random forest models differed from one city to another (Table 1). On average, wind speed (with a normalized importance value at 25%) is the most important predictor variable, followed by day_lunar (17%), relative humidity (14%), temperature (12%), year (11%), day_julian (11%), and wind direction (9.6%). The pattern of variable importance is generally consistent with the atmospheric mechanisms [2,34,35]. For example, wind fields (i.e., wind speed and wind direction) are associated with the accumulation, dispersion, and transport of air pollution plumes in the atmosphere [47,48]. Temperature is closely related to NO_2_ concentrations by affecting atmospheric turbulence and the reaction rate of atmospheric oxidants with NO_2_ [49]. Relative humidity has an impact on NO_2_ concentrations through the hydroxyl radical, which is produced by photolytic reactions involving water vapor [50].

The differences in the random forest models for different cities are very likely due to a number of influencing factors including the data characteristics and the urban design [29,34]. For instance, we used the nearest weather station to represent the meteorological conditions at the fixed-site stations, which is a common practice in air quality studies [9,32,34,40,41]. However, there are uncertainties regarding the meteorological conditions and the uncertainties may be quite different in different studied cities. In addition, the urban design (e.g., urban building morphology) also affects the dispersion and transport of NO_2_ pollution and the observed NO_2_ concentration at the fixed-site stations [22,51], which may further influence the correlation between observed and predicted NO_2_ concentrations in the studied cities.

### 3.2. Comparison between the Machine Learning and Difference-to-Difference Approaches

**Figure 2:**
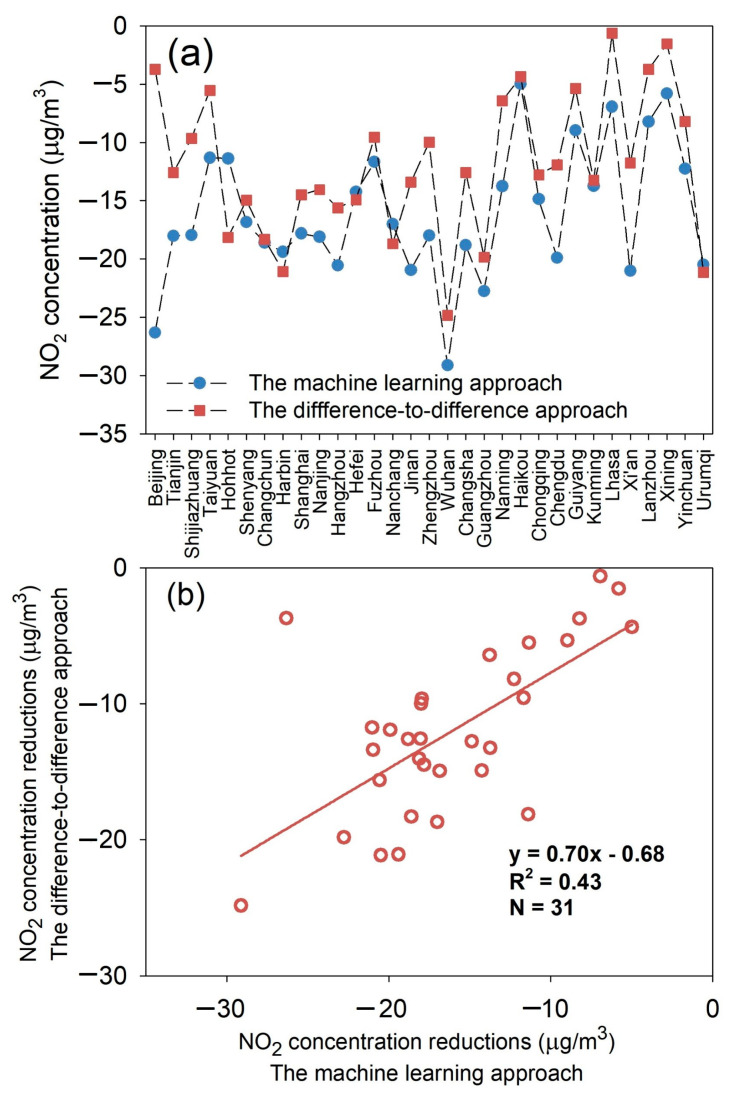
The comparison between the machine learning and difference-to-difference approaches. (**a**) The NO_2_ concentration reductions estimated by the two approaches, and (**b**) the correlations between the NO_2_ concentration reductions estimated by the two approaches.

**Figure 3:**
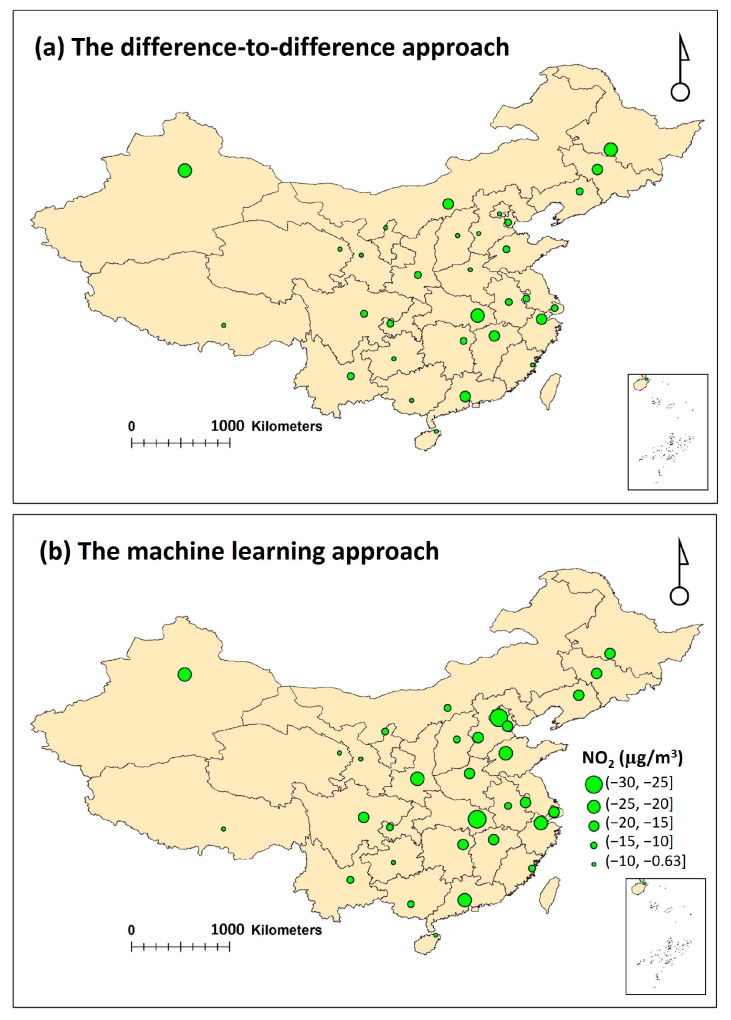
The spatial distribution of NO_2_ concentration changes due to the COVID-19 lockdown using (**a**) the machine learning and (**b**) difference-to-difference approaches. The base map is the distribution of China’s provinces, which was retrieved from the website of Resource and Environmental Science Data Platform (https://www.resdc.cn/DOI/DOI.aspx?DOIID=122 accessed on 15 October 2023).

A similar spatial pattern was found between the machine learning and difference-to-difference-derived NO_2_ concentration reductions, but the exact estimated NO_2_ concentration reduction values for each city varied between the two approaches (Figure 2 and Figure 3). Compared with the machine learning approach, the difference-to-difference approach tends to underestimate the effect of COVID-19 lockdown measures on NO_2_ pollution; these two sets of estimates were generally moderately correlated, with the R^2^ value at 0.43 (Figure 2 and Figure 3, Table 2). The meteorological conditions in the 2020 COVID-19 lockdown period prevented the dispersion of air pollution with persistent low wind speed, frequent temperature inversion, and wind convergence compared with the pre-lockdown period [52,53]. The machine learning approach captured this meteorological variability better than the difference-to-difference approach, thus resulting in the discrepancies of the estimation results. In the following analysis, only the results using the machine learning approach are presented and discussed.

### 3.3. Spatiotemporal Variability of NO_2_ Concentration Reductions over China

**Figure 4:**
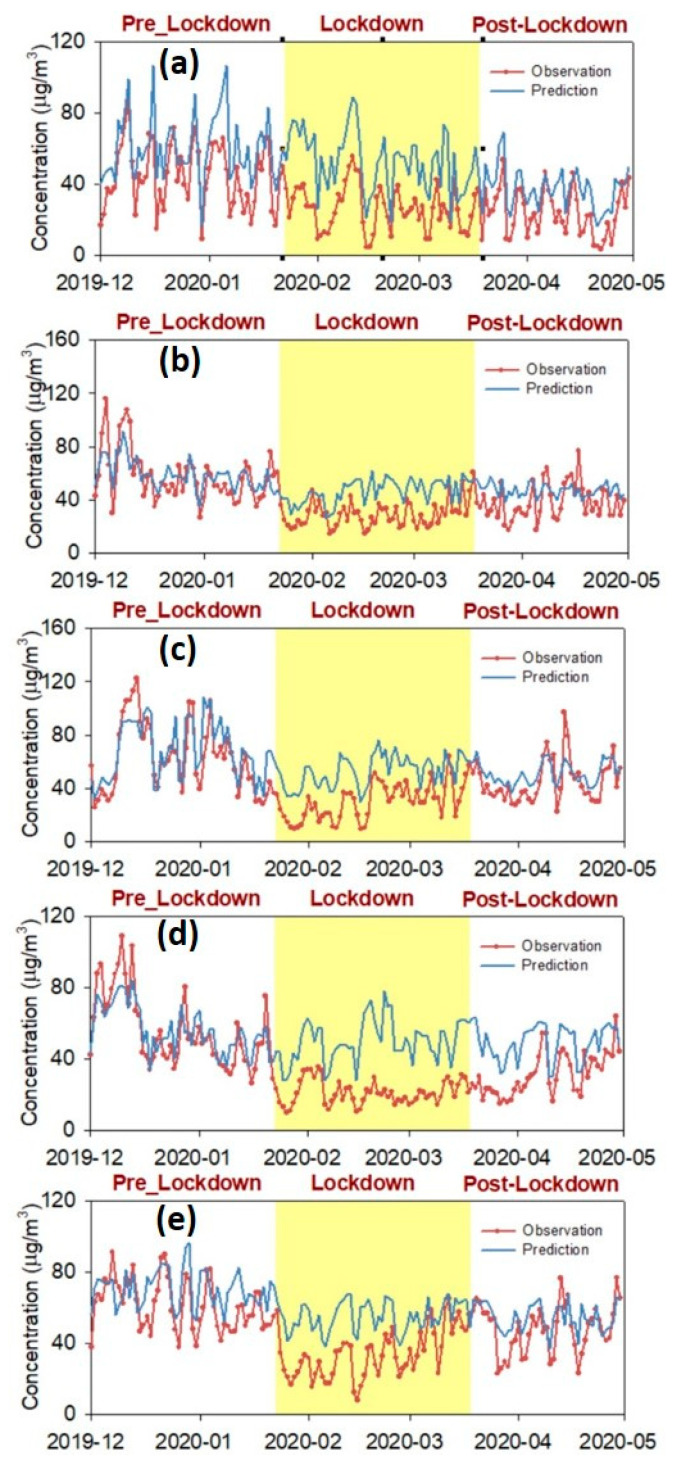
Time series of NO_2_ predictions and observations during 1 December 2019 to 30 April 2020 in five selected cities, including (**a**) Beijing, (**b**) Shanghai, (**c**) Guangzhou, (**d**) Wuhan, and (**e**) Xi’an.

As shown in Figure 4, during the pre-lockdown period, the random forest model-predicted NO_2_ concentrations generally captured the trend and most peak values of NO_2_ observations. For example, the IOA values for the pre-lockdown period was 0.72, 0.38, 0.52, 0.62, and 0.56, respectively, in Beijing, Shanghai, Guangzhou, Wuhan, and Xi’an, indicating the high predictive accuracy of the established random forest models. The time series plots confirmed this finding. There are clear gaps between the NO_2_ predictions and observations during the COVID-19 lockdown period. These differences can reasonably be inferred as the effect of COVID-19 lockdown measures on the NO_2_ pollution. During the post-lockdown period, the differences between NO_2_ predictions and observations are due to both the model uncertainty and the gradual recovery of human activities [13,14,54]. For example, there was still a clear difference between NO_2_ predictions and observations during the middle of March until the start of April in Wuhan (Figure 4). This is because the COVID-19 lockdown lasted a bit longer in Wuhan than other cities.

Due to the COVID-19 lockdown, the NO_2_ concentrations were reduced in all of the 31 studied cities, ranging from 5.0 µg/m^3^ (for Haikou) to 29 µg/m^3^ (for Wuhan). The reduction percentage varied from 15% (for Lanzhou) to 58% (for Wuhan) (Figure 2 and Figure 3, Table 2). Consistent with previous studies [7,10,24,52,55], a relatively larger reduction in NO_2_ concentration was observed in central eastern China, i.e., the Beijing–Tianjin–Hebei region, and the Yangtze River delta region. This is likely due to the fact that relatively strict lockdown measures were implemented in these areas. During the lockdown period, the urban traffic intensity and industrial activities were significantly reduced due to the reduction of human mobility [7,9,12,56].

### 3.4. Mortality Benefits from the Reduction of NO_2_ Pollution

**Figure 5:**
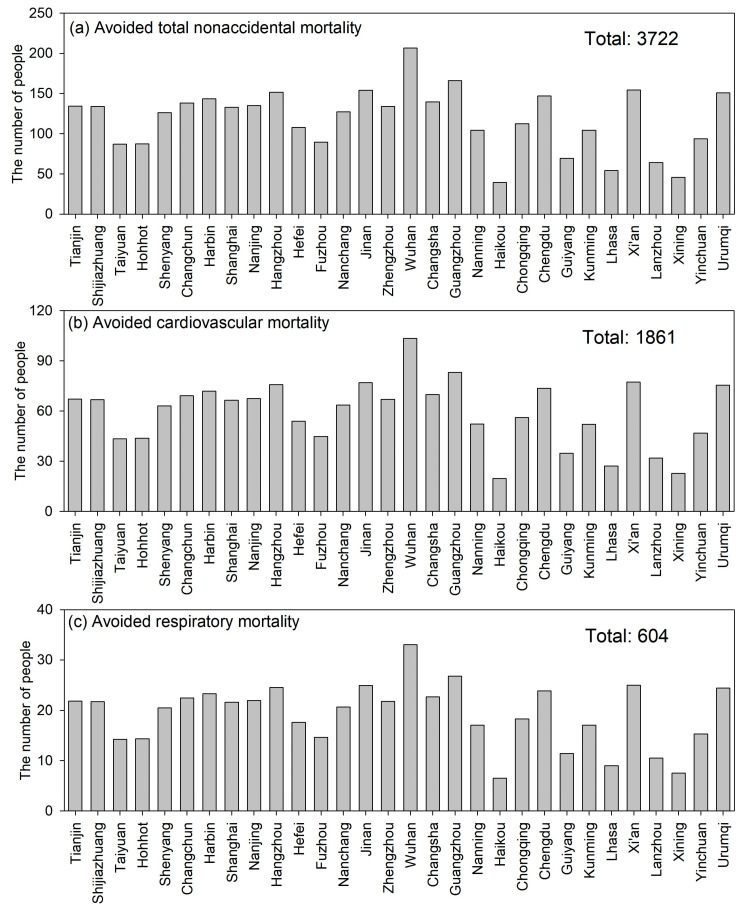
The estimated avoided disease-related deaths, including non-accidental diseases, cardiovascular diseases, and respiratory diseases.

Few studies have evaluated the human health benefits due to the air quality improvements during the COVID-19 lockdown period [57]. Our study tried to fill this gap by providing insights into the human health benefits of the COVID-19 lockdown with improved air quality. In the present work, we estimated the avoided total non-accidental mortalities, avoided cardiovascular mortalities, and avoided respiratory mortalities due to NO_2_ reductions during the COVID-19 lockdown period. The total avoided number of deaths for these three categories were 3722, 1861, and 604 (Figure 5). The number of avoided disease-related premature deaths is mainly affected by the NO_2_ concentration reduction level and the number of residents in the target cities. With the substantial reduction in NO_2_ concentrations and the larger number of residents, there is a larger number of estimated avoided disease-related premature deaths (Figure 5).

### 3.5. Atmospheric Implications

Previous studies usually only used the difference between the 2020 pollutant concentration level and the average pollutant concentration level over the previous several years to represent the effect of the COVID-19 lockdown on the air pollution [1,5,10,56,58]. For example, Chu et al. [10] found a significant reduction in NO_2_ concentrations during the COVID-19 lockdown period in 2020 compared with the 2019 level. Overall, this is a quite useful approach, but it does not take the variability of meteorological conditions into consideration [15,22,29]. The difference-to-difference approach roughly considered the variability in meteorological conditions with the pre-lockdown difference adjusted, but there is still uncertainty. The ACTMs can quantify the effect of meteorology but it is complex and time-consuming to perform these ACTM simulations [7,8,27]. In our study, the proposed random forest machine learning approach can accurately consider the year-to-year variability in the meteorology among the modelling period and it is generally easy and fast to conduct these machine learning simulations to obtain results. The ability of the proposed machine learning approach depends largely on the availability of predictor variables. The machine learning approach can be easily applied to the modelling of changes in multiple air pollutants from abrupt emission changes when the key data inputs are ready.

Our study confirmed that the sharp restrictions on human activities (e.g., reduction of traffic intensity) resulted in a direct reduction in NO_2_ pollution and associated health benefits. Several studies have reported that severe haze events with high PM_2.5_ concentrations occurred during the COVID-19 lockdown period [8,52], which is likely due to the unfavorable meteorological conditions (e.g., high humidity), the regional transport of secondary aerosols, and the uninterrupted emissions from some sources (e.g., power plants) [59]. The evolution of O_3_ during the COVID-19 lockdown period is even more complex because of the influence of meteorology and the discrepancy in its responses to the precursors [7,54]. We have tried to establish random forest machine learning models for PM_2.5_ and O_3_, but the statistical performance of the models for some cities was quite low. The possible reasons can be the quality of the datasets and the complex chemical reactions for the formation of these two air pollutants [13,29]. Take O_3_ as an example; it is formed through the chemical reactions between nitrogen oxides and volatile organic compounds under sunlight, and also affected by meteorology and regional transport [28]. In future studies, more efforts should be paid to work on the simulations of other air pollutants (e.g., PM_2.5_ and O_3_) for the design of a balanced control strategy for multiple air pollutants [59,60]. 

Our study highlights the fact that there should be a balance between the design of short-term vs. long-term air quality management. In the present study, due to the specific event of the COVID-19 lockdown, the short-term reduction in NO_2_ pollution was observed. However, with the society recovering to a normal status, the NO_2_ concentrations gradually increased to levels almost comparable with previous years (Figure 4). The rebound of NO_2_ concentrations during the post-lockdown period could provide useful insights for implementing sustainable long-term environmental policies. In the past two decades, China has implemented a set of control measures to mitigate air pollution. As a result of this, the NO_2_ concentration has decreased steadily [4]. Also, short-term emission regulations during specific events, like the APEC Blue and Parade Blue in Beijing, were successfully applied [61]. For the sustainable development of cities and society, a precise implementation of long-term air quality policies should be applied [9,62,63]. In the long-term, the air quality management policies of technological advances in vehicles and power generation, the adjustments of industry structures, and the adoption of new energy sources are recommended [1,14].

## 4. Conclusions

Compared with the difference-to-difference approach, the established random forest machine learning models accurately explained the impact of the drivers (e.g., lockdown policy and meteorology) of NO_2_ pollution. On average, it was estimated that the city-average NO_2_ concentration was reduced by 16 µg/m^3^ during the 2020 COVID-19 lockdown period, with 3722 avoided non-accidental deaths. Simulations on other cities and air pollutants using various analysis approaches should be performed to obtain a better understanding of the evolution of air quality during the COVID-19 lockdown period. The proposed methodology has the potential to be applied in other short-term emission reduction or emission-boosted cases, e.g., wildfire events or volcano eruption incidents, when the major predictor variables are available. Future studies should include datasets of satellite-based remote sensing and ACTM simulations as potential predictor variables to better capture the spatial variability of air pollution. Furthermore, other machine learning or deep learning algorithms should be tested in these types of case studies to further improve the predictive capability and understanding of variables influencing air quality.

## Figures and Tables

**Table 1:** The statistics of normalized variable importance (%) for the random forest models.

Predictor Variables	Variable Importance (%)		
	Minimum	Maximum	Mean
Year	4.4	25	11
Day_julian	7.9	17	11
Day_lunar	10	24	17
Wind direction	4.7	16	9.6
Wind speed	5.2	36	25
Temperature	7.6	16	12
Relative humidity	3.6	30	14

**Table 2:** The NO_2_ concentration reductions (µg/m^3^) due to COVID-19 lockdown estimated by the machine learning and difference-to-difference approaches.

City	NO_2_ Concentration Reductions	
	The Machine Learning Approach	The Difference-to-Difference Approach
Beijing	−26	−3.7
Tianjin	−18	−13
Shijiazhuang	−18	−9.6
Taiyuan	−11	−5.5
Hohhot	−11	−18
Shenyang	−17	−15
Changchun	−19	−18
Harbin	−19	−21
Shanghai	−18	−15
Nanjing	−18	−14
Hangzhou	−21	−16
Hefei	−14	−15
Fuzhou	−12	−10
Nanchang	−17	−19
Jinan	−21	−13
Zhengzhou	−18	−10
Wuhan	−29	−25
Changsha	−19	−13
Guangzhou	−23	−20
Nanning	−14	−6.4
Haikou	−5	−4.4
Chongqing	−15	−13
Chengdu	−20	−12
Guiyang	−9	−5.4
Kunming	−14	−13
Lhasa	−6.9	−0.63
Xi’an	−21	−12
Lanzhou	−8.2	−3.7
Xining	−5.8	−1.5
Yinchuan	−12	−8.2
Urumqi	−20	−21

## Data Availability

The data presented in this study are available on request from the corresponding author.
